# Adipokines, Myokines, and Cardiokines: The Role of Nutritional Interventions

**DOI:** 10.3390/ijms21218372

**Published:** 2020-11-08

**Authors:** Pamela Senesi, Livio Luzi, Ileana Terruzzi

**Affiliations:** 1 Department of Biomedical Sciences for Health, Università degli Studi di Milano, 20131 Milan, Italy; pamela.senesi@unimi.it (P.S.); livio.luzi@unimi.it (L.L.); 2 Department of Endocrinology, Nutrition and Metabolic Diseases, IRCCS MultiMedica, 20138 Milan, Italy

**Keywords:** cardiovascular diseases, metabolic syndrome, obesity, nutraceuticals, adipokines, myokines, cardiokines

## Abstract

It is now established that adipose tissue, skeletal muscle, and heart are endocrine organs and secrete in normal and in pathological conditions several molecules, called, respectively, adipokines, myokines, and cardiokines. These secretory proteins constitute a closed network that plays a crucial role in obesity and above all in cardiac diseases associated with obesity. In particular, the interaction between adipokines, myokines, and cardiokines is mainly involved in inflammatory and oxidative damage characterized obesity condition. Identifying new therapeutic agents or treatment having a positive action on the expression of these molecules could have a key positive effect on the management of obesity and its cardiac complications. Results from recent studies indicate that several nutritional interventions, including nutraceutical supplements, could represent new therapeutic agents on the adipo-myo-cardiokines network. This review focuses the biological action on the main adipokines, myokines and cardiokines involved in obesity and cardiovascular diseases and describe the principal nutraceutical approaches able to regulate leptin, adiponectin, apelin, irisin, natriuretic peptides, and follistatin-like 1 expression.

## 1. Introduction

The pandemic of obesity is a crucial increasing health and economic emergency. Excessive fat tissue represents a major risk factor for the development of chronic diseases, such as insulin resistance and type 2 diabetes mellitus [1] and for onset of cancer [2]. As of 2020, data related to SARS-CoV-2 infection indicates that obesity worsens COVID-19 outcomes, including death [3]. However, cardiovascular disease (CVD), especially heart failure (HF) and coronary heart disease, is the most important comorbidity associated with obesity and CVD remains the main cause of death worldwide [4].

As known, adipose tissue produces various bioactive molecules, called adipokines. An unbalanced secretion of adipokines is correlated with oxidative stress and chronic low-grade inflammation and these alterations are the key players involve in the onset and exacerbation of cardiovascular complications [5,6].

Moreover, data obtained by animal and human studies have revealed that also skeletal muscle and heart act as endocrine organs and their secreted hormones, respectively, called called myokines and cardiokines. It has been well recognized that crosstalk between these molecules regulates obesity, insulin resistance, and cardiac complications, as reviewed by several researchers [7,8,9,10]. Therefore, the identification of new therapeutic agents able to influence this network could have a substantial positive impact on obese patients and on cardiovascular damage.

There is also a growing interest in the role of caloric restriction diet and nutraceutical compounds on the regulation of expression of adipokines, myokines, and cardiokines [11].

In the last decades, the U.S. National Institute on Aging mainly investigated caloric restriction diet (CR) effects on aging. CR is a long-term dietary intervention characterized by reduced caloric intake, but malnutrition and deficiency of essential nutrients are prevented [12,13]. The health advantages of CR resulting in lifespan extension are well established in many studies [14,15]. Moreover, accumulating evidence indicates that CR represents an effective strategy to reduce weight, influences adipose tissues plasticity, and modifies endocrinological function of adipose tissue and skeletal muscle. Then, experimental data obtained by animal studied show that CR reduced the risk for several diseases, including diabetes and cardiovascular diseases [16]. In particular, CALERIE (Comprehensive Assessment of Long term Effects of Reducing Intake of Energy) clinical trial has demonstrated that 2 years of moderate CR significantly ameliorated both whole-body and regional adiposity and decreased multiple cardiometabolic risk factors in young, non-obese adults [17,18]. In any case, all molecular targets of CR are not yet identified as well as action on adipokines, myokines, and cardiokines is not yet clarified.

There is a growing body of evidence that gut dysbiosis may contribute to the development and progression of obesity and CVD, primarily increasing oxidative and inflammation state [19,20]. Based on these data, several studies have analyzed how prebiotic and probiotic supplementation could be useful to restoring gut eubiosis and consequently improve cardiometabolic state [21]. Although, many of the published data are conflicting, and several points (i.e., use of only bacterial strain, time of treatment, use of mixture) remain to be clarified in order to formulate an effective and easy nutritional intervention.

During the 1970s, a rising body of evidence provided data to support omega-3 polyunsaturated fatty acids (n-3 PUFA) supplementation in the prevention and management of obesity and CVD [22]. Even in vitro studies in cell cultures, animal models, epidemiological, and clinical studies in human subjects present conflicting data and usually there is a large gap in the literature regarding the effects observed in vitro/animals studies and in clinical trials.

However, as is well known, polyphenols have great relevance in the scientific community. Polyphenols, plant-derived natural compounds containing at least one aromatic ring and a hydroxyl group, are the most important source of antioxidants in diet. Several studies have demonstrated the relationship between diet enriched with polyphenols and cardiovascular disease [23]. Moreover, recent results have shown that the effects of polyphenols-enriched diet could improve weight loss and metabolic state. In any case, not all the results described in the literature agree regarding the efficacy or the molecular target of the nutritional intervention based on the use of polyphenols [24]. This discrepancy is probably due to different chemical forms of polyphenols (8000 different compounds are known) but above all the innumerable study designs (i.e., in vitro, in animal models, with or without exercise) the dosage of polyphenols, and the duration of the treatment.

Therefore, the purposes of this review are to (i) briefly describe the biological role in the pathogenesis of obesity and cardiac diseases of main adipokines (leptin and adiponectin), myokines (irisin and apelin), and cardiokines (natriuretic peptides and follistatin-like 1); (ii) examine how nutritional interventions modify the expression of thek04se molecules.

It is important to underline that we chose to focus our attention on four types of nutritional intervention: (i) CR because is an anti-aging diet and obesity and CVD are the main comorbidities in the elderly; (ii) prebiotics and probiotics considering the role of gut dysbiosis in the pathogenesis of CVD and obesity; (iii) 3-n PUFA whose consumption is associated with an improvement in cardiovascular risk but biological action is not yet clarified; (iv) polyphenols supplementation, considering their action as antioxidative and anti-inflammatory molecules.

## 2. Leptin

### 2.1. Leptin: Biological Role

Leptin is probably the most known adipokine. After its discovery in 1994 [25], research demonstrated that leptin is produced exclusively by adipocytes and is crucial for body weight regulation. After its binding with specific receptors in the arcuate nucleus of the hypothalamus, leptin inhibits appetite [26,27].

In animal models and humans, serum leptin levels correlate with BMI and with percentage body fat. Otherwise, obese patients are characterized by leptin “resistance” that is a reduced tissue response to leptin [27,28]. Resistance to leptin aggravates obese condition and then contributes to an additional rise in leptin levels creating a vicious cycle called “leptin-induced leptin resistance” [29,30].

Leptin high concentration improves oxidative stress and inflammation and above all cardiovascular diseases [31]. Strong data have revealed that while functioning leptin improves fatty acid β-oxidation, prevents toxic lipid accumulation, and consequently expression of oxidative and inflammation mediators, elevated leptin levels caused abnormal lipid accumulation [32,33].

At the same time, high leptin concentrations have vasoconstrictor action stimulating endothelin 1, a strong vasoconstrictor molecule. While under physiological condition, leptin supports nitric oxide release enhancing vasodilation [34]. In this manner, leptin contributes to onset of obesity associated hypertension [35,36].

Leptin signaling deficiency is correlated with a higher risk to onset of cardiac dysfunctions and heart failure [33]: in obese mice, as leptin- or leptin receptor-deficient rodent models, elevated leptin concentrations induces cardiac hypertrophy, contractile and blood pressure abnormalities [37,38]. Morphologically, elevated leptin levels are associated with hypertrophy and in diabetic patients with cardiomyopathy that display diastolic dysfunction at early stages and systolic dysfunction and reduced ejection fraction in later stages [37]. Moreover, impaired leptin signaling causes metabolic dysfunctions, mainly high leptin levels increases fatty acid oxidation and simultaneously reduces glucose uptake inducing cardiac insulin resistance and the shift from glucose to free fatty acid metabolism [38]. This metabolic switch triggers excessive lipid accumulation in cardiac cells and induces lipotoxicity, mitochondrial dysfunctions, and increased production of reactive oxygen species [37,38]. Additionally, recent data indicated that impaired leptin signaling contributes to cardiac fibrosis increasing collagen deposition and oxidative stress condition [39].

### 2.2. Leptin: Nutritional Interventions

Reliable through many studies, different diets and nutritional supplementations are able to modify leptin expression. Interestingly, calorie restriction (CR) influenced leptin secretion and increased mitochondrial function and insulin sensitivity. In particular, An et al. have observed that caloric restriction therapy ameliorates left ventricular hypertrophy in mice characterized by impaired leptin signaling [40] (Table 1).

Additionally, various studies have demonstrated that probiotic and prebiotic supplementation improves glycemic parameters and leptin concentrations in patients affect by obesity, diabetes, and non-alcoholic fatty liver disease (NAFLD) (Table 2). In obese mice, probiotic intervention using *Lactobacillus rhamnosus, Lactobacillus acidophilus,* and *Bifidobacterium bifidumi* reversed abnormalities in the gut microbiota profile and improved leptin resistance [41]. Similarly, in a mouse model of NAFLD, probiotic mixture ameliorates not only hepatic steatosis but also decreased leptin concentration and inflammatory biomarkers [42]. Behrouz et al. have observed that oligofructose dietary fiber supplementation with lifestyle intervention (exercise and diet) decreased elevated leptin values in NAFLD patients [43]. However, in diabetic patients only multistrain probiotic supplementation over 6 months ameliorates metabolic, cardiac, and inflammatory profiles without modifying leptin levels [44]. Probably, in humans administration of prebiotics and probiotics is more effective in association with lifestyle changes and future studies should aim to clarify the role of prebiotics/probiotics on leptin synthesis [45].

Additionally, the relationship between n-3 PUFA and leptin should be further investigated (Table 3). Current data indicate that in obese patients n-3 PUFA increases leptin concentration while in lean subjects PUFA decrease circulating levels of leptin [46]. However, in obese women, the combination of lifestyle changes and supplementation with n-3 fatty acids eicosapentaenoic acid (EPA) and docosahexaenoic acid (DHA) mitigates inflammatory and metabolic damages but does not influence leptin levels [47]. Conversely, in obese adolescents, omega-3 increased lifestyle interventions diminish insulin resistance, leptin concentrations, and endothelial dysfunction [48].

It is important to note that also the correlation between 3-n PUFA intake and cardio cardiometabolic disorders is not yet completely clarified and for example in obese-diabetic mice dietary-linolenic acid (ALA) enhances cardiac parameters improving inflammatory status, leptin synthesis but does not influence body weight and glycemic status [49]. In another study performed using obese mice, 3-n PUFA supplementation reduced leptin concentrations and inflammation state ameliorating cardiometabolic risk [50]. The different results could be due to different dose or timing of supplementation.

Moreover, probably the combination strategy based on lifestyle interventions and 3-n PUFA supplementation [47,48] is an effective clinical and applicable approach to control inflammatory state associated with leptin and subsequently decreases cardiovascular risk, in any case additional investigations should be performed to determinate the mixture of 3-n PUFA and the duration of treatment.

Moreover, an increasing number of studies have analyzed or polyphenolic compounds potential benefits in impaired leptin signaling and cardiovascular diseases (Table 4). For example, a combination of quercetin and resveratrol improves leptin sensitivity, promotes weight loss, mitigates metabolic dysfunction, normalizes gut microflora, and prevents cardiac damage [51]. In the same manner, some authors observed that lycopene, found in tomatoes and other red fruits and vegetables, reduces the inflammatory state in obesity decreasing hyperleptinemia [52]. In addition, a recent meta-analysis indicates that lycopene could have a cardioprotection ability improving blood lipids profile, blood pressure, and endothelial function [53]. Probably, nutritional strategies based not only on single polyphenol but on functional foods enriched with polyphenolic compounds should be validated.

Indeed, based on 3-n PUFA and polyphenols actions, some researchers studied the effect of nuts eating on leptin concentration [54,55]. Nuts contain primarily oil but also polyphenols and as known leptin-induced damage is correlated with inflammatory and oxidative stress. In obese animal fed with high carbohydrate and high-fat diet, walnut oil increases insulin sensitivity and antioxidant capacity and concurrently decreases leptin concentration [54]. Moreover, Goldwin et al. have demonstrated that supplementation with mixed nuts promotes satiety in overweight and obese adults and reduces leptin concentration [56] (Table 5).

In summary, the results described above show how different nutritional approaches influence leptin levels even if the combined effect of dietary supplementation and exercise should be clarified.

## 3. Adiponectin

### 3.1. Adiponectin: Biological Role

Another crucial adipokine involved in cardiac pathology is adiponectin (APN). Human APN is encoded by Adipo Q gene and primarily secreted by white adipocytes in three different isoforms: trimer, hexamer, and multimer [57]. A tight correlation between the complex of structure, the molecular weight, and the biological function characterized these oligomeric isoforms. Indeed, researchers have revealed that APN has both pro-inflammatory and anti-inflammatory action and then a dual role in in different pathological conditions, including cardiovascular diseases.

Recent studies have hypothesized that multimer APN has anti-inflammatory and pro-inflammatory action, while trimer APN could stimulate only pro-inflammatory signaling [58]. It is important to note that multimer and hexamer APN are the isoforms detected in human blood, while APN trimers are present at very low concentrations and consequently are not detected [58,59,60].

Primary, APN function is energy homeostasis and is known as “starvation protein” [45]. Leptin and APN have opposite roles in energy metabolism: APN enhances glucose intake and prevents gluconeogenesis and fatty acid accumulation activating AMPK signaling. Obese patients are characterized by low APN concentrations and an altered ratio between leptin and APN is associated with BMI, impaired insulin signaling, and inflammatory state [61].

Above all, reduced APN levels is closely associated with cardiovascular diseases. Using an atherosclerosis animal model characterized by APN deficiency (ApoE knock-out mice), different groups have demonstrated that endogenous treatment with APN increased anti-inflammatory genes expression, including eNOS and IL-10, inhibits pro-inflammatory genes expression, i.e., TNF-α and IL-6. Additionally, APN decreases NF-kB signaling that plays a key role in inflammatory processes and oxidative stress [62,63].

Data, obtained using this animal model and other studies, show that multimer APN acts an anti-inflammatory hormone by the activation of AMPK-GLUT4 and AMPK-eNOS pathways, that consequently neutralizes NF-kB signaling. Decreased APN concentration observed in obese patients, and the resulting AMPK inactivation, promotes not only lipid accumulation, but also chronic inflammation state induced by NF-kB [62,63]. In contrast to multimer APN, APN trimers seem to stimulate nuclear translocation of NF-kB by MAP kinases activation [64].

### 3.2. Adiponectin: Nutritional Interventions

As well as for the leptin, different works have studied the possible effects of CR on APN (Table 1). In animal obese models, CR attenuates left ventricular hypertrophy and diastolic dysfunction, increasing APN [65]. Moreover, Ding et al. have demonstrated that CR improves insulin resistance condition enhancing APN by adipocytes [66]. Additionally, in obese patients, this nutritional approach increases APN concentration promoting weight loss: the data obtained from several studies suggest that to obtain significant results 4–6 weeks of CR are sufficient [67,68]. Moreover, various evidence shows the additive benefits of the combined treatment CR and exercise on APN levels and consequently on obesity and CDV: aerobic exercise, also moderate exercise like yoga, increased CR effect on APN expression [69,70].

In addition, probiotics and prebiotics could have a possible effect on APN circulating levels (Table 2). Although few studies have been carried out aimed to analyze prebiotics action on APN, in any case Hume et al. have observed that prebiotic supplementation, formed from oligofructose-enriched inulin/d, ameliorate appetite control, and increase APN levels in children with overweight and obesity [71]. Several studies indicate that supplementation with numerous types of probiotics contributes to an improvement in APN secretion. For example, as reported before, Sabico et al. have tested multistrain probiotic supplementation in diabetic patients observing after 6 months no effect on leptin level but a significant increase of APN associated with a reduction of inflammation state and an improvement of cardiometabolic profile [44]. Additionally, Bernini et al. have demonstrated that *Bifidobacterium lactis* HN019 supplementation for 90 days improved adiponectin and nitric oxide levels increasing antioxidant defenses in patients [72]. On the other hand, another study has shown that consumption of probiotic soy milk and soy milk does not influence APN level and inflammation state in T2DM patients [73]. As mentioned above, Behrouz et al. have treated patients with probiotic supplementation, but they observed only a significant decreased in leptin levels whereas APN serum concentration remained unchanged [43]. Therefore, the above findings suggest that probiotics and, to a lesser extent prebiotic, have a modulatory action on APN, therefore, there is evidently a need for more studies that would identify which different bacterial strains are more effective, the duration of the treatment, and the dose of daily consumption of probiotics [74].

Different works reported that PUFA ingestion ameliorates APN deficiency in obese patients [75]. Moreover, Redondo Useros et al. using an animal model have demonstrated that 2-hydroxyoleic acid, synthetic hydroxylated fatty acid, decreases body weight restoring APN levels [76] (Table 3).

In agreement with the above discussion about nutritional interventions and leptin levels, antioxidant compounds could be a promising natural coadjuvant in the treatment of obesity and CVD, acting on APN (Table 4). Meta-analysis of randomized controlled trials performed by Mohammadi-Sartang et al. has revealed that resveratrol supplementation significantly improves adiponectin signaling [77]. Clark et al. have designed the same study of Mohammadi-Sartang et al. aimed to study the relationship between APN concentrations and curcumin, another well-known antioxidant compound. They observed that, as well as resveratrol, curcumin significantly enhances adiponectin levels [78].

Accumulating evidence suggests that coffee, that contains different antioxidant molecules including caffeine and catechins, is a function food. Numerous studies and clinical trials have highlighted a positive relationship between coffee consumption and APN concentrations. It is important to note that decaffeinated coffee has no effect on APN expression [79]. In vitro experiments have demonstrated that caffeine promotes APN protein synthesis activating PPARγ [80]. Additionally, coffee intake improves of glycemic parameters, inflammation state, and reduces cardiovascular risk [81,82].

Finally, Bageri et al. have investigated the effect of a treatment based on exercise and supplementation with green tea extract, which like coffee contains caffeine and catechins, observing a rise of APN and above all, that this combination mitigates metabolic abnormalities and counteracts inflammatory state [83].

In general, results obtained by studies carried out using single molecules having antioxidative action or function food suggest that this nutritional intervention contributes to restore APN levels in obese patients and to improve metabolic condition and cardiovascular risk.

## 4. Apelin/APJ Axis

### 4.1. Apelin/APJ Axis: Biological Role

The Apelin/APJ pathway is an emerging and promising therapeutic target for treatment of cardiovascular disease, insulin resistance, and type 2 diabetes [84].

The APJ receptor, first identified in 1993, is a member of the G protein-coupled receptor family closely related to the angiotensin receptor, but it is not coupled by angiotensin II [85,86]. Only in 1998 was an APJ endogenous ligand found: apelin (APLN) [87].

APLN is a hormone secreted specifically by hypothalamus, but it is widely expressed in various organs, including fat tissue, heart, and skeletal muscle and, therefore, APLN is an adipomyokine. Bioactive APLN is produced by enzymatic cleavage of proapelin, 77 animo acid protein. Now, several isomers of proapelin are known and all forms are biologically active, including apelin-36 and apelin-13 [84,87].

Recent evidence highlights how APLN has many biological functions and above all plays a crucial role in the cardiovascular and metabolic systems [84,86,88].

In detail, data obtained by animal and human studies have demonstrated that APLN positively regulates blood pressure promoting the vasodilative effect of nitric oxide, secretion of arginine vasopressin-AVP (antidiuretic hormone) and counteracting the vasoconstrictive effect of angiotensin II and the sodium retention [89,90,91,92,93]. Xie H et al. demonstrated by meta-analysis study that reduced APLN level was significantly associated with an increased risk for hypertension development [94].

However, above all, APLN has a protective role in heart failure, including acute pathologies like myocardial infarct and chronic heart disorders, such as cardiac hypertrophy [89,95,96,97].

Using animal models, some research observed that exogenous APLN treatment reduced infarct size and simultaneously increased heart rate [95,96,98] while apelin-knockout mice are characterized by increased myocardial infarction size and mortality [99,100].

Data reported in literature reveal that apelinergic signaling plays a crucial role in decreased ROS production [101] and protection the heart against ischemia/reperfusion injury activating Akt, ERK/MAPK, and eNOS pathways [102,103].

However, some authors have reported proinflammatory action of APLN [104]: in a clinical study performed by Heinonen et al. patients with metabolic syndrome showed high level of APLN and TNF-α [105] while other studies revealed that APLN mitigates hepatic macrophage infiltration and TNF-α expression and reduces myocardial inflammation induced by diabetes condition or sepsis shock [106,107,108,109].

Other exciting discoveries in recent years has been the evidence that APLN/APJ pathway is fundamental in aging: senescence is characterized by lower levels of APLN/APJ and this alteration is associated with impairment of cardiac contractility but above all with inflammation and oxidative stress [110,111]: Apelin treatment alleviates aging damage as demonstrated by Yang et al. and Rai et al. [112,113]. Interestingly, numerous data reveal that APLN amplifies exercise benefits: in old mice model and in humans, exercise improving APLN expression ameliorates skeletal and cardiac dysfunctions and moreover exercise-induced APLN improves cognitive functions [114,115,116].

In obese animal models, APLN improves metabolic state [117,118]: Bertrand et al. have demonstrated that APLN increased glucose uptake and insulin sensitivity [119].

However, some data reported in literature show that APLN level is increased in obese patients suggesting that BMI is positively correlated with APLN levels [120,121]. Moreover, some authors have suggested that in obese patients APLN expression is elevated in white adipose tissue and decreased in brown adipose tissue. Furthermore, recent investigations suggest that APLN enhances browning process of white adipocytes [122]. In view of these data, the relationship between APLN/APJ signaling and adipose tissue plasticity and between APLN/APJ should be clarified by other investigations.

### 4.2. Apelin/APJ Axis: Nutritional Interventions

Growing bodies of data indicate that APLN/APJ axis is positively involved in cardioprotection processes and counteracts cardiac and metabolic damage induced by aging. Treatments with synthetic apelin peptides or nutritional strategies aimed at improving the APLN/APJ system may represent an important therapeutic option for cardiovascular disease. Considering the recent discovery of this adipomyokine, research evaluating the correlation between nutritional interventions and APN are limited.

To our knowledge, few studies have evaluated the effects of caloric restriction diet or similar diet and APLN. After low-calorie diet APLN concentration is reduced in obese subjects [123] and recent evidence indicates that not total carbohydrate intake, but dietary glycemic index and load are correlated with APLN expression [124]. Krist et al. have studied the action of 6 months calorie-restricted diet in obese patients observing significantly reduced APLN serum concentrations. Moreover, reduced APLN levels significantly relate to improvement of insulin resistance [125]. Ramadan fasting, a particular caloric restriction diet, is not associated with changes in Apelin-13 levels in 13 healthy men, as observed by Celik et al. [126] (Table 1).

Very few data reported probiotics action on APLN levels, in 44 women affected by polycystic ovary syndrome, of which insulin resistance is the most important pathophysiologic characteristic, 12-week synbiotic supplementation decreases Apelin-36 serum concentration [127] (Table 2).

In addition, omega-3 fatty acids supplementation in patients with cardiovascular diseases increased APLN levels, decreases inflammation state, analyzed through sensitive C-reactive protein, and ameliorates lipid profile. Additionally, eicosapentaenoic acid supplementation increases APLN/APJ expression in muscle and adipose tissues improving insulin signaling in lean and obese mice [128,129] (Table 3).

Moreover, experimental studies have proved potential therapeutic actions of polyphenol compounds on APLN levels: curcumin supplementation mitigates APLN abnormalities in diabetic rats and in obese patients [130] (Table 4).

Considering the importance of the APNL/APJ axis on cardiovascular and metabolic disease, the nutritional role is promising, and then further studies are necessary.

## 5. Irisin

### 5.1. Irisin: Biological Role

In 2012 Böstrom et al. identified a new mitokine: irisin that is usually secreted after exercise [131,132].

Irisin is a proteolytic product of fibronectin type III domain-containing protein 5 (FNDC5) that is activated by PGC1-α [133]. More recent data reveal that irisin is an adipomyokine because irisin is also secreted by white adipose adipocytes [134]. Biological action on irisin is correlated to obesity and cardiovascular disease.

Irisin improves energy expenditure stimulating the browning of white adipose tissue by enhancing expression of UCP1 in adipocytes [134]. For this reason, irisin is a thermogenic protein and other data indicate that irisin is an insulin sensitizing hormone. Indeed, irisin promotes glucose uptake by skeletal muscles and lipid metabolism [135,136]. Further, irisin plays an important role in hepatic metabolism reducing lipogenesis and gluconeogenesis and increasing lipid oxidation and glycolysis [137,138]. These actions are principally mediated by AMPK activation and calcium [139,140]. Then, considering irisin as a thermogenic and insulin sensitizing molecule, several studies investigated the possible correlation between circulating irisin and adiposity, but the results are controversial: some authors observed a positive correlation between irisin levels and BMI whereas others reported a negative correlation [140]. This aspect requires additional investigations.

Moreover, in patients affected by NAFLD, irisin is able to decrease hepatic oxidative stress and mitigate inflammation state [141]. Therefore, irisin is an anti-inflammatory/oxidative molecule: this adipomyokine inhibits expression of pro-inflammatory cytokines, including TNF-α, and IL-6 in a concentration dependent manner and decreases migration of macrophages in adipose tissue [142].

The positive action of irisin is not limited to fat and hepatic tissue. In heart, irisin improving mitochondrial functions protects cardiomyoblasts from various injuries characterized by elevated ROS production, i.e., ischemic and reperfusion [143], high glucose-induced [144], and lipotoxic-induced injury [145]. Moreover, human different reports have shown that irisin levels were lower in the diabetic patients with cardiovascular complications [146] and in subjects with myocardial infarction or heart failure [147]. Finally, in a mouse model of cardiac ischemia, irisin treatment reduces infarct size, promotes neo angiogenesis, and enhances the reestablishment of cardiac function [148].

### 5.2. Irisin: Nutritional Interventions

Based on data about irisin biological action, nutritional strategy aiming to improve irisin signaling could be useful to mitigate obesity and cardiac conditions.

In literature, there are few data about the possible action of caloric restriction diet on irisin expression. Using a mouse model, Shirvani et al. have observed that primarily aerobic exercise and not caloric restriction diet influences irisin expression [149] (Table 1).

Indeed, as previously reported [132,133], irisin secretion is stimulated by exercise and for this reason some authors have investigated the possible synergic action between exercise and nutrition supplementation. For example, Batitucci et al. have observed that taurine supplementation combined with a high intensity physical training improves irisin levels in obese women [150]. In the same manner, Eskandari et al. have recently investigated the effects of interval jump rope exercise combined with dark chocolate supplementation in obese adolescents observing an increased irisin levels after 6 weeks [151]. The propensity to investigate the combined action of exercise and physical activity represents a limit to clarify nutritional effects on irisin expression.

Kwon et al. have investigated only the action of *L. plantarum* treatment in mice fed high fat diet, observing that this treatment enhances browning and thermogenesis of adipose tissue increasing APN and irisin expression [152] (Table 2).

As well as leptin and adiponectin, the effect of 3-n PUFA on irisin was studied in diabetes subjects: 3-n PUFA supplementation increases irisin expression [153] (Table 3).

Probably the nutraceuticals that have been most studied as modulators of irisin expression are polyphenols. In mice fed with genistein, an important isoflavone, browning process is facilitated by increasing of irisin concentrations [154]. Additionally, Kheiripour et al. irisin improving metabolic condition in the liver of diabetic rats [155]. Additionally, in patients affected by NAFLD green cardamom, rich in polyphenols, mitigates lipid accumulation, insulin signaling is impaired, and inflammatory state increases irisin levels [156]. Furthermore, grape pomace extract, a functional food containing numerous polyphenols and antioxidative molecules, promotes browning of white adipose tissue improving irisin signaling in obese rats [157].

Considering that numerous clinical studies have reported that vitamin D supplementation mitigates cardiovascular disease [158,159], it is worth mentioning that over the years various groups have observed a link between vitamin D and irisin expression. In diabetic animal models, vitamin D increases irisin expression [160]. In overweight and obese patients, depletion of vitamin D is associated with worsened inflammatory state in obese [161], whereas vitamin D stimulus upregulated PGC-1α expression and consequently irisin secretion [162] Moreover, Safarpour et al. have demonstrated that in obese and diabetic subjects vitamin D supplementation counteracts impaired insulin signaling and increased irisin signaling pathway [163]. Then, the relationship between irisin and vitamin D should be further investigated.

## 6. Natriuretic Peptides

### 6.1. Natriuretic Peptides: Biological Role

The main endocrine hormones released from the heart are atrial natriuretic peptide (ANP) and B-type natriuretic peptide (BNP), that have an analogous protein structure formed by a peptide ring with a cysteine bridge. Like other hormones, the active form of these cardiokines are the proteolytic products of long inactive peptides and their secreted by cardiomyocytes in response to stress conditions and mechanical stretch [164].

Both ANP and BNP play a crucial role in preserving cardiovascular homeostasis and promoting defense mechanisms against the adverse impacts of volume and pressure overload. It is well established that ANP expression is induced by elevated atrial pressures, whereas BNP is secreted in response to a reflection of ventricular overload. Kidney represents the main target organ of these cardiokines and they stimulate diuresis and natriuresis and consequently ANP and BNP promotes vasodilatation counteracting blood pressure abnormalities [165,166].

In patients affected by heart failure, elevated levels of ANP and BNP relate with disease severity and have a negative prognostic value. Moreover, using animal models several groups have observed that ANP and BNP are involved in myocardial ischemia and hypoxia regulating inflammation response [164,165,166].

However, in the last years, different clinical studies have revealed that altered concentrations of natriuretic peptides characterized obese patients: the presence of low circulating levels of BNP and ANP is typical in obesity and indeed this condition is known as “natriuretic handicap”. As reported before, plasma natriuretic peptides levels were also described to be negatively correlated with cardiometabolic diseases and they are a predictive value in the development of diabetes mellitus [167,168].

Accumulating evidence underlines that ANP and BNP induces lipid metabolism and adipose tissue remodeling enhancing mobilization of lipid in white adipose tissues, energy dissipation in brown adipocytes, and above all these cardiokines improve the browning process [169,170]. Of interest, ANP and BNP modify expression and secretion of adiponectin, natriuretic peptides are positively associated with adiponectin [171].

Then, natriuretic peptides act on whole-body fatty acids metabolism and mitigate the impaired insulin pathway and consequently ameliorate glucose homeostasis, insulin sensitivity, and consequently cardiometabolic state [172].

### 6.2. Natriuretic Peptides: Nutritional Interventions

To our knowledge, few data about the influence of caloric restriction diet and ANP/BNP are reported in literature. In any case, it is noteworthy that exercise activities improve ANP and BNP expression whereas Western diet and high fat diet decrease natriuretic peptides secretion [173]. In the future it could be interesting to investigate this aspect also considering that caloric restriction reduces cardiovascular risk [174,175]. Moreover, considering the physiological role of irisin and APLN, deepening the relationship between these adipomyokines and natriuretic peptides could be interesting.

Gan et al. have investigated in rat the effects of *Lactobacillus rhamnosus* GR-1 administration [176]. In rats an myocardial infarction was induced and after 6 weeks of supplementation the authors have detected an reduction of myocardial hypertrophy associated with decreased ANP levels [176] (Table 2). In the same manner, probiotic-fermented purple sweet potato yogurt supplementation improves cardiac hypertrophy downregulating ANP and BNP expression in hypertensive rat hearts [177] (Table 5).

Moreover, in 2016 Wang et al. have performed an important meta-analysis of randomized controlled trials in order to clarify the role of 3-n PUFA in heart failure concluding that 3-n PUFA decreases BNP levels. Then, 3-n PUFA could be useful in heart failure but in any case many aspects are yet to clarified from time of supplementation to the dosage and components of 3-n PUFA [178] (Table 3).

As reported for adipokines and myokines, different polyphenolic derivatives regulate natriuretic peptides expression. In in vitro and animal studies, resveratrol mitigates cardiac fibrosis improving anti-inflammatory mechanics and downregulating ANP and BNP gene expression [179]. In the same manner, another antioxidative nutraceutical compound, curcumin alleviates cardiac hypertrophy and decreases ANP and BNP gene expression [180]. Different works suggests the use of curcumin as therapeutic adjuvant strategies in the management of cardiac damage induced by hyperglycemia; indeed in animal models this molecule improves not only antioxidative pathways but also insulin sensitivity [181,182,183] (Table 4).

As reported before, vitamin D could play an important role in cardiac damage. Different clinical trials have observed that in heart failure patients, vitamin D supplementation improves cardiac function and quality of life acting on BNP levels [184]. Moreover, in animal models, vitamin D reduces myocardial fibrosis or cardiac hypertrophy attenuating ANP and BNP expression [185,186]. These results are promising but addition animal and clinical studies should be performed, aiming to identify the dose of vitamin D and the timing of treatment.

## 7. Follistatin-Like 1

### 7.1. Follistatin-Like 1: Biological Role

Follistatin-like 1 (FSTL-1) is a glycoprotein secreted by cardiomyocytes and skeletal myocytes. It is characterized by a partial homology to the follistatin family [187]. Several findings reveal that FSTL-1 is a cardioprotective cardiokine involved in cardiac ischemic response and remodeling [188,189,190].

Data obtained by animal models indicate that FSTL-1 has protective action on ischemia/reperfusion injury preventing cardiomyocytes apoptosis [190,191]. Moreover, FSTL-1 expression decreases cardiac hypertrophy in in vitro and in vivo models [192]. Recent evidence suggests that FSTL-1 promotes antioxidative signaling in heart increasing expression of nuclear factor (erythroid-derived 2)-like 2 (Nrf2), a crucial factor involved in oxidative response [193]. FSTL-1 could represent a novel biomarker for cardiac inflammatory and oxidative stress responses.

Recently, Xu et al. have investigated in obese mice the possible role of FSTL-1 as adipokine. They have discovered that FSTL-1 protein expression in the adipose tissue and obese mice are characterized by higher FSTL-1 protein levels compared to lean mice. This result corroborates data obtained by human analysis that have shown how circulating FSTL-1 levels in obese and diabetic subjects were higher than in lean subjects. Moreover, Xu X et al. have demonstrated that FSTL-1 protein expression increased dramatically in response to physical activity in healthy subjects [194]. These results confirm study performed by Kon et al. that have proved that acute endurance exercise may stimulate the secretion of FSTL1 and apelin [195]. Moreover, Fang et al. suggest that FSTL-1 promotes brown adipose thermogenesis [196].

### 7.2. Follistatin-Like 1: Nutritional Interventions

Very few studies have analyzed the effects of different nutritional interventions or compounds on Follistatin-like 1. Jafari Salim et al. have, recently, investigated the possible PUFA supplementation on FSTL-1 protein expression: in a small group of patients with coronary artery disease PUFA treatment increased FSTL-1 and decreased inflammation state [197] (Table 3).

Considering the new data, FSTL-1 is a cardio/adipokine and then it could be a new target for therapeutic nutritional interventions for obesity and related cardiovascular diseases. Probably, probiotics and prebiotics could be useful; indeed data reported in literature show that FSTL1 expression is correlated with lipopolysaccharide, a potent endotoxin involved in gut dysbiosis [198,199]. Further investigations should be performed.

## 8. Discussion

An important area to focus on in future metabolic and cardiac research involves the identification of relevant nutritional interventions having a positive action on adipokines, myokines and cardiokines synthesis. As summarized in Table 1, Table 2, Table 3 and Table 4, caloric restriction and probiotic and prebiotic supplementation, 3-n PUFA, and polyphenols could be a promising therapeutic option in the future. However, many problems still need to be resolved.

As reported in tables, in animal or human studies rarely adipokines, myokines, and cardiokines are simultaneously evaluated: usually only leptin and adiponectin are measured together. This approach limits understanding the action of different nutritional interventions.

The lack of an overall picture could partly explain the different results that are often observed in animal and in human studies testing the same treatment. Clinical trials are often extremely disappointing, and the beneficial effects observed in animals are not found in humans. As known, patient compliance is a limit in clinical studies. Currently, the clinical nutritional studies foresee that subjects/patients independently eat a specific supplement or follow a particular diet. Participants do not always adhere to diet programs and in addition dietary and exercise diaries are not correctly compiled. In the future, these problems could be solved using “apps” that allow continuous monitoring [200].

Moreover, the pharmacokinetics of nutritional compounds and the route of administration represent another problem: for example, the half-life and the rate by which the supplement needs to be taken are usually different in rodents and humans [200].

Importantly, genetic aspects should be considered: as now nutrigenomics is important as well as nutrigenetics and inter-individual variation is a crucial component of human clinical studies [200].

As previously reported, another criticism is represented by physical exercise. An obese and or cardiopathic subject is advised to carry out aerobic activity but physical activity modifies the expression not only of myokines but also of other molecules such as adiponectin. Consequently, exercise could be a confounding element in evaluating the effectiveness of nutritional interventions. However, considering the importance of exercise it could be appropriate to always test a specific nutritional intervention in association with exercise and not consider the two aspects individually.

Additionally, the dose and type of prebiotics/probiotics, 3-n PUFA, and above all polyphenolics to be used is a critical aspect. As reported in Table 2, Table 3 and Table 4, numerous polyphenolic compounds were tested as well as several 3-n PUFA and probiotics. A possible solution to simplify nutritional investigations could be to focus attention on studying the effects of functional foods. These foods often contain more nutrients, such as nuts rich in both 3-n PUFA and polyphenols, and are easier to administer and could also be enriched with additional nutraceutics, such as yogurt with antioxidants. Table 5 shows some functional foods, presented in this review, that are capable of modifying the expression of adipokines, myokines, and cardiokines [52,53,79,81,83,156,157,177]. Therefore, the action of functional foods will have to be further investigated.

It is important to point out that in this review we analyzed nutritional intervention in animals or in childhood or adult individuals affected by obesity, diabetes, and/or CVD. However, as is known, maternal nutrition and breastfeeding play a fundamental role in delineating gut microbiota, in obesity and cardiovascular diseases onset. Recently, various working groups have studied the action of nutritional supplementation in pregnant or breastfeeding women [201,202]. For example, leptin and adiponectin are important components of breast milk and several data have shown that adipokines, breast milk adipokines, influence phenotype in adulthood [203,204] and then studying the effect of nutritional supplementation on breast milk adipokines and myokines and cardiokines is more interesting. Pomar et al. have recently evaluated the impacts of maternal intake of an unbalanced food intake during lactation observing that breast milk leptin and adiponectin levels were greater while levels of irisin were lower [205]. This is an important finding of this study and further research is needed to clarify the interaction between maternal intake and breast milk adipokines, myokines, and cardiokines levels.

Finally, as it will be extremely important to analyze the relationship between breast milk composition and maternal diet, it could be important to study the effects of the various nutritional interventions in lean subjects. These nutritional interventions could promote the maintenance of optimal state of health and to prevent obesity and CVD onset. Above all, it could be extremely interesting to evaluate the effectiveness of these nutritional interventions in middle-aged individuals, who are characterized by an increased risk of developing obesity and cardiovascular disease [206].

## 9. Conclusions

**Figure 1:**
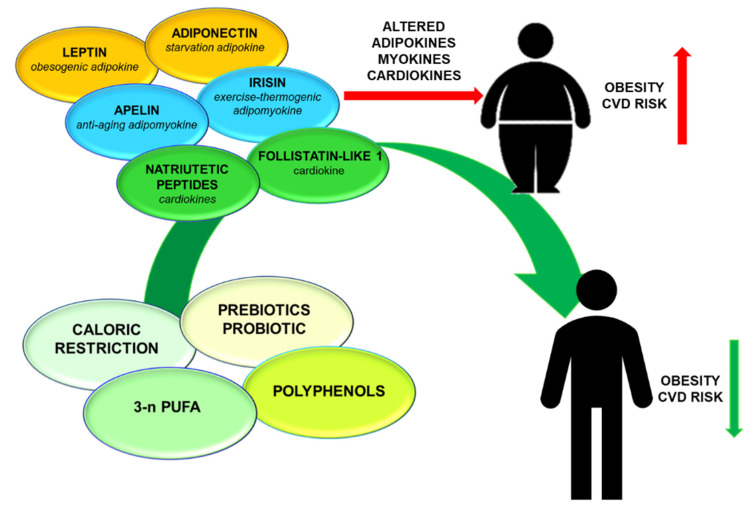
Schematic summary of nutritional interventions on adipokines, myokines, and cardiokines Obesity and consequently cardiovascular diseases are characterized by an altered secretion of adipokines, myokines, and cardiokines. In this review, nutritional interventions (caloric restriction, prebiotic or probiotic supplementation, 3-n PUFA, and polyphenols) having a positive action on these molecules, are described. These nutritional interventions could ameliorate cardiometabolic state in obese subjects.

The discovery of endocrinological function of adipose tissue, skeletal muscle, and heart significantly modified the paradigms of obesity and cardiovascular pathogenesis. Adipokines, myokines, and cardiokines play a fundamental role in regulating metabolic state and cardiac function. Obese condition and cardiovascular diseases are characterized by an altered secretion of these molecules. Caloric restriction, prebiotic or probiotic supplementation, 3-n PUFA, and polyphenols represent emerging and promising strategies to influence endocrinological function of adipose tissue, skeletal muscle, and heart (Figure 1). The definition of nutritional interventions, that could promote a healthy lifestyle acting on adipokines, myokines, and cardiokines, will represent an exciting challenge for future research.

## Figures and Tables

**Table 1:** Effects of caloric restriction on adipokines, myokines, and cardiokines.

Biological Action	Adipo-myo-cardiokines	Type of Study
↓left ventricular hypertrophy	↓leptin resistance	Obese mice ^a^
↓myocardial function	↑adiponectin levels	Obese mice ^b^
↓weight loss	↑adiponectin levels	Rats ^b^
↑body composition, ↑metabolic parameters	↑adiponectin levels	Obese and overweight former athletes ^b^
↑liver steatosis	↑adiponectin levels	Obese mice ^b^
↑resting metabolic rate	↑adiponectin levels	Obese or overweight women ^b,^*
↑insulin sensitivity	↑apelin expression and levels	T2DM patients ^c,^*
↑metabolic state	= apelin levels	Healthy adult men ^c^
↑aerobic exercise	↑irisin levels	Obese rats ^d,^*

^a^ See Section 2.2; ^b^ see Section 3.2; ^c^ see Section 4.2; ^d^ see Section 5.2; * in association with exercise.

**Table 2:** Effects of prebiotic and probiotic supplementation on adipokines, myokines, and cardiokines.

Type of Intervention	Biological Action	Adipo-myo-cardiokines	Type of Study
*Lactobacillus rhamnosus, L. acidophilus, Bifidobacterium bifidumi*	↑insulin sensitivity	↓leptin resistance	Obese mice ^a^
Probiotic mixture	↓liver steatosis inflammatory state	↓leptin levels	Mouse model of NAFLD ^a^
Oligofructose dietary fiber + lifestyle modifications	↑metabolic condition	↓leptin levels = adiponectin levels	Patients with NAFLD ^a^
Multistrain probiotic	↑metabolic and cardiac state ↓ inflammatory state	=leptin levels ↑adiponectin levels	T2DM patients ^a^
Oligofructose-enriched inulin/d	↑satiety	↑adiponectin levels	Children with overweight and obesity ^b^
*Bifidobacterium lactis* HN019	↑nitric oxide levels	↑adiponectin levels	Subjects with and without the metabolic syndrome ^b^
Probiotic soy milk	↑lipid profile	=adiponectin levels	T2DM patients ^b^
Synbiotic	no significant beneficial metabolic effects	↓apelin levels	PCOS women ^c^
*L. plantarum*	↑browning and thermogenesis of adipose tissue	↑irisin levels	Obese mice ^d^
*Lactobacillus rhamnosus* GR-1	↓myocardial hypertrophy	↓ANP levels	Rats after myocardial infarction ^e^

Legend: ^a^ see Section 2.2; ^b^ see Section 3.2; ^c^ see Section 4.2; ^d^ see Section 5.2; ^e^ see Section 6.2.

**Table 3:** Effects of 3-n PUFA supplementation on adipokines, myokines, and cardiokines.

Type of Intervention	Biological Action	Adipo-myo-cardiokines	Type of Study
EPA-DHA + lifestyle modifications	↓inflammatory state ↑metabolic state	=leptin levels	Obese women ^a^
Omega-3 + lifestyle modifications	↓insulin resistance ↓endothelial dysfunction	↓leptin levels	Obese adolescents ^a^
Linolenic acid	↑cardiometabolic parameters ↓inflammatory state	↓leptin levels	Obese-diabetic mice ^a^
3-n PUFA	↑cardiometabolic state ↓inflammatory state	↓leptin levels	Obese mice ^a^
	↑insulin sensitivity, improve blood pressure	↑irisin levels	T2DM patients ^b^
	↑heart failure	↓BNP levels	Meta-analysis of randomized controlled trials ^c^
	↓inflammatory state	↓follistatin-like 1 levels	Patients with coronary artery disease ^d^
2-hydroxyoleic acid (2-OHOA) and n-3 PUFA	↑body composition ↑cardioprotection mechanims	↑adiponectin levels	Obese mice ^e^
EPA	↑insulin signaling	↑apelin levels	Lean/obese mice ^f^
	↑insulin signaling in adipose tissue	↑apelin levels	Lean/overweight rats ^f^

Legend: ^a^ See Section 2.2; ^b^ see Section 5.2; ^c^ see Section 6.2; ^d^ see Section 7.2; ^e^ see Section 3.2; ^f^ see Section 4.2.

**Table 4:** Effects of polyphenolic supplementation on adipokines, myokines, and cardiokines.

Type of Intervention	Biological Action	Adipo-myo-cardiokines	Type of Study
Combination of quercetin and resveratrol	↑body composition ↓ inflammatory state and gut dysbiosis	↑leptin levels	Obese mice ^a^
Lycopene	↓inflammatory state	↓leptin expression and levels	Obese mice ^a^
Resveratrol	↑body composition	↑adiponectin levels, = leptin	Meta-analysis of randomized controlled trials ^b^
	↓cardiac fibrosis ↓inflammatory state	↓ANP and BNP levels	Cardiomyocytes (in vitro) ^c^
Curcumin	↑body composition	↑adiponectin levels	Meta-analysis of randomized controlled trials-obese mice ^d^
	↑insulin sensitivity	↓apelin levels	T2DM rats ^e^
	↓cardiac hypertrophy and fibrosis	↓ANP and BNP levels	Rats ^f^
	↓cardiac hypertrophy oxidative stress	↓ANP and BNP levels	Diabetic rats ^f^
Genistein	↑browning of adipose tissue	↑irisin levels	Adipocytes-mice ^g^
Silymarin	↑hepatic condition ↑anti-oxidative mechanisms	↑irisin levels	T2DM rats ^g^

Legend: ^a^ See Section 2.2; ^b^ see Section 3.2; ^c^ see Section 6.2; ^d^ see Section 3.2; ^e^ see Section 4.2; ^f^ see Section 6.2; ^g^ see Section 5.2.

**Table 5:** Effects of function foods on adipokines, myokines, and cardiokines.

Type of Intervention	Biological Action	Adipo-myo-cardiokines	Type of Study
Walnut oil	↑insulin sensitivity ↑antioxidative capacity	↓leptin levels	Obese rats ^a^
Mixed nuts	↑satiety	↓leptin levels	Overweight and obese adults ^a^
Coffee	↓inflammatory state	↑adiponectin levels	Women with or without type 2 diabetes ^b^
	↑body composition ↑metabolic state	↑adiponectin levels	Diabetic rats ^b^
Green tea extract + exercise	↑metabolic state ↓inflammatory state	↑adiponectin levels	Overweight middle-aged men ^b^
Green cardamom	↑liver steatosis and insulin signaling ↓inflammatory state	↑irisin levels	Overweight or obese with NAFLD ^c^
Grape pomace extract	↑browning of adipose tissue	↑irisin levels	Obese rats ^c^
Probiotic-fermented purple sweet potato yogurt	↓cardiac hypertrophy	↓ANP and BNP levels	Hypertensive rat ^d^

Legend: ^a^ See Section 2.2; ^b^ see Section 3.2; ^c^ see Section 5.2; ^d^ see Section 6.2.

## Abbreviations

ALA	Linolenic acid
ANP	Atrial natriuretic peptide
APLN	Apelin
APN	Adiponectin
BNP	B-type natriuretic peptide
CR	Caloric restriction diet
CVD	Cardiovascular disease
DHA	Docosahexaenoic acid
EPA	n-3 fatty acids eicosapentaenoic acid
FSTL-1	Follistatin-like 1
n-3 PUFA	Omega-3 polyunsaturated fatty acids
NAFLD	Non-alcoholic fatty liver disease
ROS	Reactive oxygen species
